# Moderately trained male football players, compared to sedentary male adults, exhibit anatomical but not functional cardiac remodelling, a cross-sectional study

**DOI:** 10.1186/s12947-021-00263-0

**Published:** 2021-11-11

**Authors:** Jan E. Engvall, Meriam Åström Aneq, Eva Nylander, Lars Brudin, Eva Maret

**Affiliations:** 1 Department of Clinical Physiology and Department of Health, Medicine and Caring Sciences, Linkoping University, Linkoping, Sweden; 2 CMIV – Center for Medical Image Science and Visualization, Linkoping University, Linkoping, Sweden; 3 Department of Clinical Physiology, Kalmar County Hospital and Department of Health, Medicine and Caring Sciences, Linkoping University, Linkoping, Sweden; 4 Department of Clinical Physiology, Karolinska Institutet, and Karolinska University Hospital, Stockholm, Sweden

**Keywords:** Fitness, oxygen uptake, ventricular volume, deformation analysis, cardiac remodelling, echocardiography, magnetic resonance imaging

## Abstract

**Background:**

Elite athletes have been the subject of great interest, but athletes at an intermediate level of physical activity have received less attention in respect to the presence of cardiac enlargement and/or hypertrophy. We hypothesized that playing football, often defined as demanding less endurance components than running or cycling, would still induce remodelling similar to sports with a dominating endurance component.

**Methods:**

23 male football players, age 25+/− 3.9 yrs. underwent exercise testing, 2D- and 3D- echocardiography and cardiac magnetic resonance (CMR). The results were compared with a control group of engineering students of similar age. The athletes exercised 12 h/week and the control subjects 1 h/week, *p* < 0.001.

**Results:**

The football players achieved a significantly higher maximal load at the exercise test (380 W vs 300 W, *p* < 0.001) as well as higher calculated maximal oxygen consumption, (49.7 vs 37.4 mL x kg^− 1^ x min^− 1^, *p* < 0.001) compared to the sedentary group. All left ventricular (LV) volumes assessed by 3DEcho and CMR, as well as CMR left atrial (LA) volume were significantly higher in the athletes (3D-LVEDV 200 vs 154 mL, CMR-LVEDV 229 vs 185 mL, CMR-LA volume 100 vs 89 mL, *p* < 0.001, *p* = 0.002 and *p* = 0.009 respectively). LVEF and RVEF, LV strain by CMR or by echo did not differentiate athletes from sedentary participants. Right ventricular (RV) longitudinal strain, LA and right atrial (RA) strain by CMR all showed similar results in the two groups.

**Conclusion:**

Moderately trained intermediate level football players showed anatomical but not functional cardiac remodelling compared to sedentary males.

**Supplementary Information:**

The online version contains supplementary material available at 10.1186/s12947-021-00263-0.

## Background

Athlete’s heart, defined as a physiological cardiac adaptation to exercise [1], has been extensively studied in elite athletes with the main findings of enlargement of all cardiac chambers and a proportionate increase in wall thickness and thus in left ventricular mass (LVM) [2, 3]. This “cardiac remodelling” is more likely to occur after a long exposure to increased hemodynamic stress, can be observed in adolescents [4] and can be observed after a few months of training in dogs [5]. It is less clear whether functional measurements such as ejection fraction and global strain remodel [4, 6]. Individual fitness is objectively measured from a maximal cardiopulmonary exercise test (CPET) where maximal oxygen consumption (VO_2 max_) is determined. Various sports are categorized in terms of the size of the endurance component of exercise [7]. Playing football is generally less demanding in terms of oxygen transport capacity than long-distance running or cycling. Still however, football has been shown to induce remodelling similar to sports with a dominating endurance component [8, 9]. Most previous studies on cardiac adaptation in football players have not measured the actual aerobic capacity of the subjects, which is relevant since e.g. national differences between type of football and training regimes exist. Furthermore, elite athletes have been the subject of great interest, but athletes at an intermediate range of physical activity have received less attention in respect to the presence of cardiac enlargement and/or hypertrophy. We investigated a team of male football players participating in the Swedish level 2 national football league and compared with a reference population recruited among sedentary male engineering students. Our primary aim was to study whether volumetric and/or functional remodelling was present and detectable with echocardiography as well as with cardiac magnetic resonance (CMR) and if that could be related to the level of physical fitness.

## Methods

### Subjects and design

**Fig. 1:**
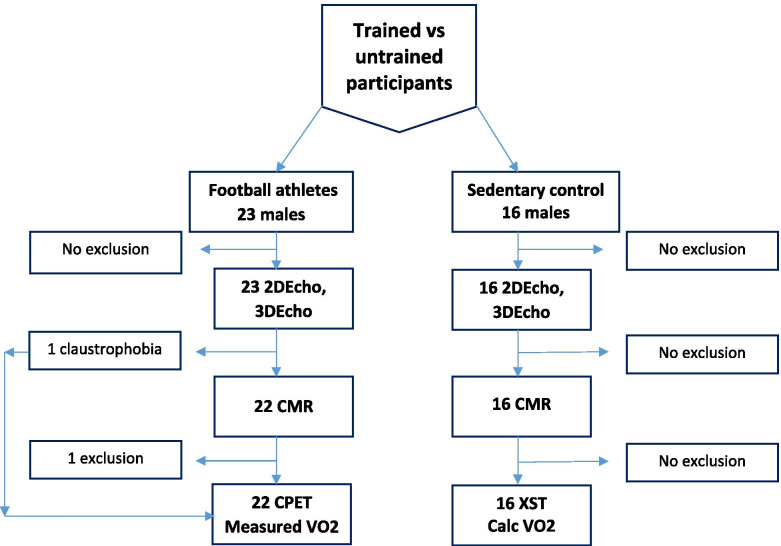
Flow chart depicting study subjects and controls

23 male football players, age 25 + 3.9 yrs., (range 18–31) were recruited from their preparticipation health screening at the beginning of the football season and were offered a comprehensive cardiac evaluation. The control group consisted of 16 male engineering students of matching age, 23 + 3.2 yrs. (range 19–31), recruited from the local University. Inclusion criterion for both groups was absence of a history of cardiac or other chronic diseases, and in addition for the control group, absence of exercise on a regular basis (cut-off less than 2 h/week). Both groups completed a questionnaire regarding background factors and smoking habits. For additional basic demographics, see Table 1. Exclusion criteria were those that could possibly interfere with the cardiac magnetic resonance examination (CMR) such as arrhythmia, the presence of a pacemaker and claustrophobia. A flow chart depicting the inclusion process is shown in Fig. 1.

**Table 1:** Subject details

	Athletes	Controls	Difference
Parameter			***p***-value
N	23	16	
**Demography**			
**Age (years)**	25 (18–31)	23 (19–31)	0.329
**Weight (kg)**	79 (67–93)	85 (55–110)	0.128
**Height (m)**	1.81 (1.65–1.95)	1.87 (1.70–1.96)	0.061
**Body mass index (kg/m2)**	24.1 (21.9–26.1)	24.4 (19.0–31.5)	0.877
**Body surface area (m2)**	2.00 (1.78–2.25)	2.12 (1.63–2.43)	0.043
**Exercise per week (hours)**	12 (8–20)	1 (0–4)	< 0.001
**Heart rate at rest (beats/min)**	58 (47–85)	71 (59–82)	0.007
**Systolic blood pressure at rest (mmHg)**	130 (110–150)	130 (120–160)	0.612
**Bicycle exercise test**			
**Max load (Watts)**	380 (300–440)	300 (210–380)	< 0.001
**Max heart rate (beats/min)**	184 (169–203)	190 (171–203)	0.012
**Calc max oxygen consumption (L/min)**	3.98 (3.09–4.64)	3.19 (2.16–3.98)	< 0.001
**Calc max oxygen consumption (mL/kg/min)**	49.7 (43.0–56.7)	37.4 (29.0–47.4)	< 0.001

Values are median and interquartile range

Each participant underwent two-dimensional echocardiography (2DEcho), three-dimensional echocardiography (3DEcho), exercise testing (CPET), and CMR, all within the same day. Immediately after the 2DEcho exam, volumetric analysis by 3DEcho was performed. 3D volumes were acquired without echo contrast agent. Echocardiography always preceded the exercise test while CMR, if occurring after exercise, always was preceded by at least 20 min of rest.

### Exercise test

The athletes performed a maximum CPET in a seated position on a cycle ergometer CASE Stress Test System 6.61 (GE Healthcare, Milwaukee, WI, USA) [10]. Cycling began with 6 min of steady-state work at a load of 100 W, followed by increases in load of 30 W/min with the goal of reaching maximal exercise capacity within 8–12 min of cycling with increasing load. Respiratory gases were collected using an oral mask and analysed breath-by-breath using Jaeger Oxycon Pro (Vyaire Inc., Mettawa, IL, USA). We calculated VO_2 max_ using the values measured during the last 60s of exercise and expressed the results in terms of mL * min^− 1^ and mL * kg^− 1^ * min ^− 1^. We considered the participants to have achieved maximal exercise capacity if the respiratory exchange ratio was > 1 continuously for 3 min or longer, which was attained by all athletes. The control group performed a similar exercise protocol. Since for their part, VO_2 max_ could not be measured, it was calculated from the attained exercise level and the peak heartrate [11], Table 1 and Additional file 1. In the following, for all calculations and presentations regarding cardiac dimensions volumes and function in relation to oxygen uptake, we have used the *calculated* VO_2 max_ in order to use the same method for athletes and controls. The only presentation of the *measured* VO_2 max_ is in Additional file 1.

### Transthoracic echocardiography

Standard transthoracic echocardiography was performed according to then current guidelines using a Vivid 7 scanner (GE Healthcare, Horten, Norway) with a 3.5 MHz M5S ultrasound probe for 2DEcho and a 3 V-D transducer for the acquisition of 3D images [12]. Two-dimensional parasternal measurements of LV septal (IVSd) and posterior wall (PWTd) thickness as well as end-diastolic LV cavity dimension (LVEDd) were measured and LVM calculated using the Devereux formula [13]. Relative wall thickness in diastole (RWTd) was calculated [12, 14]. Apical four- (4Ch) and two-chamber (2Ch) views were recorded and LV volumes (LVEDV, LVESV), stroke volume (LVSV) and ejection fraction (LVEF) were calculated using the modified biplane Simpson formula. Analysis was performed offline using EchoPac BT 12 (GE Healthcare, Horten, Norway).

LV global longitudinal strain (LVGLS) was analysed from three apical views (4 Ch, 2Ch and the apical longaxis view, “3Ch”) using speckle tracking. LA strain was calculated on a global level, based on the relative reduction of the LA circumference in the 4Ch view [15].

Right ventricular (RV) end-diastolic dimension (RVEDd) was measured in the focused four-chamber view at the base of the RV and fractional area change (RVFAC) was calculated after delineation of the cavity in end-diastole and end-systole. Systolic displacement of the RV tricuspid annulus (TAPSE) was measured using M-mode positioned in the lateral annulus. Right atrial area was measured in the modified apical 4-Ch view and RA strain was calculated based on the relative reduction of the RA circumference.

A pulsed-wave Doppler with a sample volume of 5 mm was recorded at the tip of the mitral leaflets in the apical 4Ch-view and the early (E) and late (A) diastolic blood flow velocities were registered and measured. Tissue Doppler early diastolic velocity was recorded at the base of the LV septum and lateral wall in the apical 4Ch-view, and a mean of the two velocities was calculated (e´). The E/e´-ratio was calculated as part of the evaluation of LV diastolic function [16]. Body surface area (BSA) was calculated and dimensions and volumes were indexed when needed.

4D-volumes of the left ventricle and the left atrium (LA) were obtained from an apical probe position. Four consecutive electrocardiographically gated beats were acquired during apnea. We analyzed the data with 4D-Auto-LVQ included in the Echopac software which also provides measurements of sphericity [17]. Additional movie files 2 and 3 show this in more detail.

### CMR acquisition protocol and analysis

CMR was performed on a 1.5 T scanner (Siemens Avanto, Siemens Healthcare, Erlangen, Germany) equipped with a 6-element phased array body matrix coil combined with 6-elements in the spine coil. Retrospectively ECG-gated images were acquired in supine position during repeated breath-holds. For cine imaging, a turbo- FLASH (fast low angle shot) sequence was used and after scout images, long-axis slices in the 4Ch-, 2Ch- and apical long-axis views were acquired as well as multiple parallel short-axis views, covering both ventricles from base to apex. No gadolinium contrast was given.

Analysis was performed on a workstation equipped with Segment v2.0 R5201 semiautomatic software for cardiac volumetric analysis [18]. Volumes and EF for both ventricles were measured after semi-automatic contouring of the short-axis stack of slices, excluding the papillary muscles, see Additional file 4. LVM was calculated from segmentation of the endo- and epicardial surfaces of the LV, Additional file 4. Papillary muscle volume was excluded from LVM. RV and LV atrioventricular plane displacement (AVPD) was assessed as well as peak RV lateral displacement (RVlat), based on CMR, by an automatic tracking algorithm implemented in the Segment software [19]. Global systolic LV circumferential strain (LVGCS) was calculated from feature tracking using Tomtec 2D-CPA-MR v2 (Tomtec GmbH, Germany), Additional file 5, while systolic RV longitudinal strain in the RV-4Ch view was measured using the Segment software, Additional file 6 [20, 21]. We used absolute numbers to describe strain, denoting − 22% longitudinal strain as a “greater” strain value than − 20% longitudinal strain, according to current recommendations [22].

### Statistical analysis

Continuous variables are presented as median and interquartile range, categorical data as frequencies. Due to the skewed distribution in some of the data points, the non-parametric Mann-Whitney U-test was used for all statistical comparisons of continuous variables between the groups. Differences between proportions were calculated with the Chi-2-test. A probability level of < 0.01 was assumed statistically significant for descriptive data with many comparisons, but for testing the main hypothesis, *p* < 0.05 was considered significant. Multiple logistic regression with backward deletion was performed for the interaction between LVEDV, RVEDV and LAESV by CMR. ICC was calculated with Medcalc® (MedCalc Software, Mariakerke, Belgium), all other statistics used Statistica v. 13 (Statsoft Inc. Tulsa, OK, USA).

### Reproducibility

Interobserver variability was determined for 3D-LVEDV by echo and for LVEDV as well as for RV-strain by CMR using the intraclass correlation coefficient and according to Dahlberg [23].

## Results

### Participant characteristics

Descriptive data of the study population are reported in Table 1. The control subjects were somewhat younger (2 yrs), heavier (6 kg) and longer (6 cm) compared to the football players, but there were no statistical differences between the groups (*p* = 0.329, *p* = 0.128 and *p* = 0.061 respectively). This also applies to body mass index (BMI 24.1 vs 24.4, *p* = 0.88) and body surface area (BSA 2.00 vs 2.12 m^2^, *p* = 0.043). In agreement with the inclusion criteria, the control subjects exercised only 1 h per week vs the athletes 12 h/week, *p* < 0.001. The controls had a higher heart rate at rest before the exercise test (71 * min^− 1^ vs 58 * min^− 1^, *p* = 0.007), and during echocardiography (see Tables 1 and 2) but no difference was found in systolic blood pressure at rest (130 mmHg vs 130 mmHg, *p* = 0.612).

**Table 2:** Echocardiography

	Athletes	Controls	Difference
Parameter			p-value
N	23	16	
Heart rate (beats * min-1)	60 (41–79)	72 (57–100)	< 0.001
**Septum**_**d**_ **(mm)**	10 (7–12)	9 (7–11)	0.044
**LVED**_**d**_ **(mm)**	56 (46–68)	53 (47–61)	0.055
**LVPWT**_**d**_ **(mm)**	9.0 (7.0–11.0)	8.0 (6.0–10.0)	< 0.001
**LVM (g)**	225 (138–268)	168.5 (103–231)	< 0.001
**LVED**_**d**_**-A4Ch (mm)**	57 (47–67)	53 (45–60)	0.005
**LA-area (cm**^**2**^**)**	20.0 (15.4–24.8)	17.5 (12.5–24.1)	0.005
**RA-area (cm**^**2**^**)**	20.0 (12.1–30.9)	16.0 (10.5–21.9)	< 0.001
**3D-LAvolume (mL)**	73 (49–106)	51 (35–88)	< 0.001
**RVIT1-A4CH (mm)**	38 (21–45)	34 (29–45)	0.053
**TAPSE (mm)**	27 (17–33)	26 (22–28)	0.009
**RVFAC (%)**	49 (40–58)	47 (37–55)	0.086
E-velocity (m * s^**−1**^**)**	0.8 (0.6–1.2)	0.8 (0.5–1.2)	0.933
A-velocity (m * s-1)	0.4 (0.3–0.5)	0.5 (0.4–1.1)	< 0.001
**EA-ratio**	2.1 (1.5–3.0)	1.4 (0.9–2.3)	< 0.001
e-prim-mean (m * s-1)	0.17 (0.11–0.24)	0.17 (0.10–0.19)	0.746
**E-eprim-ratio**	5.1 (0.0–6.3)	5.0 (0.0–7.6)	0.682
**2D-LVEDV (mL)**	176 (128–239)	150 (112–178)	< 0.001
**2D-LVESV (mL)**	78 (53–114)	63 (41–77)	< 0.001
**2D-LVEF (%)**	56 (49–64)	57 (53–67)	0.329
**3D-LVEDV (mL)**	200 (145–245)	154 (117–195)	< 0.001
**3D-LVESV (mL)**	80 (60–128)	67 (40–93)	0.003
**3D-LVSV (mL)**	117 (79–148)	91 (68–107)	< 0.001
**3D-LVEF (%)**	59 (47–67)	58 (50–66)	1.000
**2D-Echo-tissue-volume ratio**	1 (1–1)	1 (0–1)	0.434
**2D-LVGLS (%)**	−19 (−24--15)	− 19 (− 22--14)	0.362
**3D-Sphericity**	0.45 (0.33–0.73)	0.33 (0.24–0.48)	< 0.001

Values are median and interquartile range

The football players had a higher frequency of family risk factors for cardiovascular disease such as diabetes and hypertension, but no one of the participants had any known history of hypertension, diabetes, valve disease or other cardiovascular disease. Two of the football players and four of the control subjects reported using bronchodilators to combat asthma. No one in this study was an active smoker, but three of the football players were using chewing tobacco/snuff.

All subjects were in sinus rhythm. There was no significant difference between the two groups regarding QRS duration (109 ms vs 102 ms, *p* = 0.20) or QRS axis (77^0^ vs 66^0^, *p* = 0.24^.^), but six of the football players compared to two in the control group fulfilled the Sokolov-Lyon criteria for left ventricular hypertrophy.

### CPET

The football players achieved a significantly higher maximal load at the exercise test (380 W vs 300 W, Table 1, *p* < 0.001) as well as higher calculated VO_2 max_ (49.7 vs 37.4 mL * kg^− 1^ * min^− 1^, *p* < 0.001) compared to the sedentary group, but peak heart rate did not differ between groups (184 * min^− 1^ vs 190 * min^− 1^, *p* = 0.012). In the football players, calculated VO_2 max_ was lower than measured, mean difference − 0.3 + 0.3 l/min, *p* < 0.001, regression equation: calculated VO_2 max_ * kg^− 1^ *min^− 1^ = 0.95x − 1.254, r = 0.79, R^2^ = 0.63 (Additional file 1 at 10.48360/zf9r-j510, [24]).

### LV and RV dimensions and volumes by echocardiography and CMR

LV and RV volumes as well as LVM are reported in Tables 2 and 3.

**Table 3:** Magnetic resonance (CMR)

	Athletes	Controls	Difference
Parameter			p-value
N	22	16	
**LVEDV (mL)**	229 (162–308)	185 (155–246)	0.002
**LVESV (mL)**	112 (69–149)	90 (66–121)	0.001
**LVEF (%)**	56 (43–64)	57 (52–62)	0.435
**LVSV (mL)**	137 (100–198)	116 (92–142)	0.003
**LVM (g)**	128 (83–152)	96 (71–120)	< 0.001
**RVEDV (mL)**	227 (177–331)	196 (157–264)	0.006
**RVESV (mL)**	96 (60–135)	79 (50–122)	0.036
**RVEF (%)**	58 (53–66)	58 (53–68)	0.891
**RVSV (mL)**	138 (114–211)	113 (90–143)	0.001
**LVAVPD (mm)**	15 (11–19)	15 (9–18)	0.569
**RVAVPD (mm)**	20 (13–27)	20 (13–24)	0.492
**RVLAT (mm)**	26 (17–34)	26 (21–32)	0.529
**LVGCS_Tomtec (%)**	−31 (−38--23)	− 31 (−42--27)	0.680
**RVGLS_Segment (%)**	−20 (− 22--17)	−18 (− 26--9)	0.693
**LA-4Ch-strain (%)**	−25 (−33--18)	−26 (− 39--18)	0.609
**RA-4Ch-strain (%)**	− 22 (− 31--10)	− 24 (− 33--13)	0.284
**LA-volume (mL)**	100 (61–134)	89 (49–117)	0.009

Values are median and interquartile range

All LV volumes (end-diastolic, end-systolic and stroke volume) assessed by 3DEcho and CMR (3D-LVEDV 200 vs 154 mL, CMR-LVEDV 229 vs 185 mL, 3D-LVESV 80 vs 67 mL, CMR-LVESV 112 vs 90 mL, 3D-LVSV 117 vs 91 mL, CMR-LVSV 137 vs 116 mL *p* < 0.001, *p* = 0.002, *p* = 0.003, *p* = 0.001, p < 0.001 and p = 0.003 respectively) were significantly higher in the athletes. The shape of the LV expressed as the 3DEcho sphericity index, differed significantly between the two groups, being higher in the athletes, 0.45 vs. 0.33, *p* < 0.001, indicating a more spherical end-diastolic shape. However, CMR could not confirm this finding. LVM and relative wall thickness (RWTd by 2DEcho) were higher in the athletes (134 g vs. 99 g, *p* < 0.001, 0.34 vs. 0.30, *p* = 0.008 respectively) while the LVM-to-volume ratio was borderline higher, 0.50 g/mL vs. 0.47 g/mL, *p* = 0.032. Three of the football players had an end-diastolic septal thickness on echo of 12 mm, but no one in the sedentary group. No study person had a calculated LVM, as assessed by CMR, higher than the recommended upper level of normal, 184 g or 91 g * m^− 2^ [25], but 11 study persons, all football players, had a calculated indexed LVM > 115 g * m^− 2^ on 2DEcho which is the upper limit of reference for LVM by linear measurements [14].

RV end-diastolic and end-systolic volumes and stroke volume were larger in the athletes, Table 3 (CMR-RVEDV 227 vs 196 mL, CMR-RVESV 96 vs 79 mL, CMR-RVSV 138 vs 113 mL, *p* = 0.006, *p* = 0.036 and *p* = 0.001 respectively). There was no difference in the left-to-right end-diastolic ventricular volume ratio between the groups (1.04 vs. 1.06, *p* = 0.936). In univariate analysis adjusted for age and height, all three o f LVEDV, RVEDV and LAESV by CMR correlated significantly with calculated VO_2 max_. In multivariate analysis, only LVEDV remained significant indicating a high interdependence between these volumes.

### LV and RV function by echocardiography and CMR

Strain parameters, EF and AVPD for the LV and the RV are reported in Tables 2 and 3. AVPD for both ventricles did not differ between athletes and controls for CMR but 2DEcho TAPSE was borderline higher for athletes (CMR-LVAVPD 15 vs 15 mm, CMR-RVLAT 26 vs 26 mm, 2DEcho TAPSE 27 vs 26 mm, *p* = 0.569, *p* = 0.529 and *p* = 0.009). Mean values for 2DEcho, 3DEcho and CMR LVEF, CMR RVEF and 2DEcho FAC were not significantly different for the athletes compared to the controls. Furthermore, LV strain by CMR in the longitudinal or circumferential directions, longitudinal LV strain by 2DEcho or RV longitudinal strain did not differentiate athletes from sedentary participants, Table 3.

### Atrial size and function

Biplane CMR LA volume was 100 vs 89 mL, *p* = 0.009 (Table 3) and with 3DEcho, 73 vs 51 ml, *p* < 0.001 (Table 2). LA- and RA-strain by CMR both showed similar results in the two groups (Table 3).

### LV and RV volume and function in relation to fitness

**Fig. 2:**
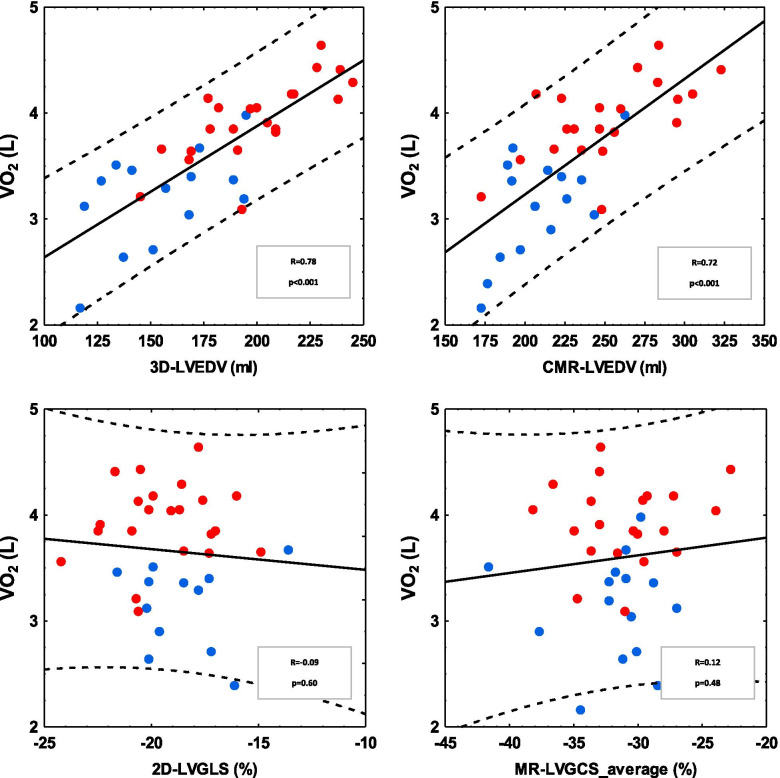
Correlation between aerobic capacity expressed as calculated VO_2 max_ and left ventricular end-diastolic volume (LVEDV) and left ventricular function expressed as left ventricular strain (LVGLS = left ventricular global longitudinal strain, LVGCS = left ventricular global circumferential strain). Red symbols = football players, blue = controls. Volumes shown in upper panels, strain in lower. Echo variables to the left and CMR to the right

Calculated maximal oxygen uptake in the combined group correlated linearly with 3DEcho-LVEDV and CMR-LVEDV, R = 0.78 and R = 0.72, Fig. 2. Furthermore, LA-volume correlated positively with calculated VO_2 max_, R^2^ = 0.39. On the contrary, there was no correlation between calculated VO_2 max_ and 2DEcho LVGLS (R = 0.09, *p* = 0.60) and with CMR-LVGCS, R = 0.12, *p* = 0.48.

### Reproducibility

LVEDV by 3DEcho calculated by two observers showed for all participants a mean value of 176 mL and a coefficient of variation (COV) 8.15% according to Dahlberg [23]. LVEDV by CMR had a mean value of 225 mL and COV 6.60%. CMR-RV global strain was − 19.0% and COV 3.49%. The corresponding ICC values for single measurements were ICC = 0.86, ICC = 0.86 and ICC = 0.94.

## Discussion

In this moderately trained group of football players, we found anatomical remodelling of the left and right ventricle as well as of the left and right atrium. Ventricular function parameters, such as EF, AV-plane displacement, longitudinal strain (echo) or circumferential strain (CMR) did not differ significantly between the football players and controls.

The volume load of endurance exercise is a stimulus to the excentric hypertrophy seen in sports with a high dynamic component [26, 27]. Previous categorization of sports in terms of their propensity to induce cardiac remodelling has been questioned and newer studies put more emphasis on the individual training intensity and duration, expressed as fitness that can be objectively measured in a maximal cardiopulmonary exercise test allowing the quantification of maximal oxygen consumption, VO_2 max_ [28]. Earlier studies have shown a close correlation between the level of fitness and cardiac chamber sizes as well as LVM [29] which we also found in this study, further supported by findings in linear LV-dimensions.

As the training literature so far has focused mainly on elite athletes, we found it of interest to elucidate the type and extent of cardiac adaptation seen in moderately trained football players which constitute a rather large athletic population in many countries. In a meta-analysis of studies measuring VO_2 max_ in football players, a relationship between the competitive level of the athletes and VO_2 max_ was found [30]. Elite football players had a relative maximal oxygen uptake between 57 and 63 mL/kg/min which is similar to that reported in an older review, 60–65 mL/kg/min [31]. The football players in our study were not on a national elite level. They were training on average 12 h/ week and their average VO_2 max_ was 49 mL/kg/min. In a large Scandinavian study, Norwegian male football players from the elite and first division league, underwent echocardiography, focusing on dimensions [9]. It revealed larger dimensions of all four cardiac chambers, indexed for BSA, compared to control subjcts. LV mass was also elevated, whereas LVEF did not differ between athletes and controls. Other functional parameters were not described in that study. Muir et al. (1999) also reported higher LV dimensions and mass in elite football players compared to controls [32]. Systolic functional parameters were not described, E/A was significantly higher in the athletes, however, a lower heart rate most probably contributed to this. Later, Akova et al. (2005) described echocardiographic structural and functional measurements in 12 basketball and 20 football players, compared to sedentary controls [33]. Average VO_2 max_ in football players was 56 mL/kg/min, LV dimensions and mass were elevated compared to controls, E-velocity or E/A ratio did not differ, neither did the myocardial performance index.

In the present study we used both echocardiography and CMR to characterize remodelling, with principally similar differences between athletes and controls, although the dimensions and volumes were not numerically equal. Echocardiography has in previous studies consistently shown lower ventricular volumes than CMR and cardiac CT, which has been regarded to be due to a tendency to favour inclusion of trabeculae in the thickness of the LV wall [34, 35]. Since chamber volumes are somewhat larger in CMR than in 3DEcho and 2DEcho and calculated LVM somewhat smaller when compared with 2DEcho, mainly due to the effects of calculation algorithms used in 2DEcho, method specific reference values are necessary to apply. 3DEcho and the use of contrast in 2D and 3DEcho have provided chamber quantification results closer to that provided by CMR [34, 36] .

Systolic functional measurements, i.e. ejection fraction, GLS (2DEcho) and GCS (CMR) did not differ between the athletes and their controls in the present study. Measurement of tissue velocity using pulsed Tissue Doppler as well as deformation analysis (strain) based on speckle tracking have been shown to be more sensitive for subtle changes in LV and RV systolic function than ejection fraction, also in athletes [6, 37]. However, we could not corroborate significant differences in LV-, RV- and LA-strain between groups and we could not find a significant correlation between aerobic capacity and strain measurements. From a meta- analysis of LV strain in athletes, Beaumont et al. concluded that data are heterogeneous regarding GLS [38]. There are however studies in high- performing endurance athletes showing lower (less negative) GLS compared to less trained or untrained subjects [39]. In the present study, absolute strain values differed between methods such as MR feature tracking GCS and 2DEcho GLS, but this is expected since GCS and GLS measure different directions of deformation and methods for feature tracking and speckle tracking echocardiography differ [20]. In a recent CMR study, Starekova et al. reported higher LV volumes and mass in football players than controls, and also lower LV and RV longitudinal strain as well as LV radial strain [40]. The aerobic capacity was not measured in that study but the football players were reported to be professional athletes.

To summarize, football players on a moderate competitional level, training on average 12 h/week (various training modalities) had an average VO_2 max_ 49 mL/kg/min which was significantly higher than in an untrained control group. The football players had significantly larger heart chamber dimensions and higher LV mass than controls. Systolic ventricular function parameters did not differ significantly between athletes and controls. Whether the type of athletic activity or the training dose is the main explanation to this remains to be proven. It has been suggested that an improved diastolic LV function is related to changes in systolic function parameters in endurance athletes. This is, however, also difficult to evaluate since many diastolic function measures are heart rate dependent which is seldom sufficiently taken into account.

Our data provide information about the degree of cardiac enlargement that may occur in moderately trained football players, which may be valuable when evaluating athletes with suspected heart disease or screened by echocardiography or CMR. It is important to measure the aerobic capacity of the athletes for an optimal definition of normality.

### Limitations

VO_2 max_ was measured in the athletes to characterize their aerobic capacity, but for comparisons between athletes and control subjects, the calculated VO_2 max_ was used in both groups.

## Conclusion

Even moderate physical training such as practiced in an intermediate level football team, induces cardiac anatomical but not functional remodelling. Oxygen uptake should be taken into consideration when assessing cardiac size even in moderately trained individuals. This study adds valuable insight into training effects in athletes at an intermediate training level, as defined from oxygen uptake measurements.

## Supplementary Information

**Additional file 1.** Computation of regression equation between measured VO2max and calculated VO2max, developed in the group of athletes.

**Additional file 2.** Segmentation and calculation of volumes and ejection fraction based on 3D echocardiography volume in an athlete. Note the large LVEDV and the higher sphericity value compared to the control in Additional file 3.

**Additional file 3.** Segmentation and calculation of volumes and ejection fraction based on 3D echocardiography volume in a control participant. Note lower LVEDV than for the athlete in Additional file 2.

**Additional file 4.** Supervised automatic segmentation of left ventricular MR slices for volumes, ejection fraction and left ventricular mass, using Segment. Cineloop shows tracking of the endo- and epicardium over the cardiac cycle.

**Additional file 5.** Automatic segmentation and calculation of circumferential LV strain using feature tracking in the 2D-Cardiac Performance Analysis software. Cine-loop shows tracking of the endo- and epicardium over the cardiac cycle.

**Additional file 6.** Tracking and calculation of RV global longitudinal and wall strain using feature tracking in Segment. Note the much higher GLS value in the RV free wall compared to the septum.

## Data Availability

The data that support the study findings are available from the corresponding author upon reasonable request. The underlying data for the calculated VO2 vs measured VO2 (Additional file 1) can be publicly accessed as a document published at Linkoping University Electronic press, 10.48360/zf9r-j510.

## Abbreviations

AVPD: Atrio-ventricular plane displacement
BMI: Body mass index
BSA: Body surface area
CMR: Cardiac magnetic resonance imaging
COV: Coefficient of variation
CPET: Cardiopulmonary exercise testing
FLASH: Fast low-angle short sequence
ICC: Intra-class correlation coefficient
IVSd: Interventricular septal diameter in diastole
LA: Left atrium
LAESV: Left atrial end-systolic volume
LV: Left ventricle
LVM: Left ventricular mass
LVEDd: Left ventricular end-diastolic diameter
LVEDV: Left ventricular end-diastolic volume
LVESV: Left ventricular end-systolic volume
LVEF: Left ventricular ejection fraction
LVGCS: Left ventricular global circumferential strain
LVGLS: Left ventricular global longitudinal strain
PWTd: Posterior wall thickness in diastole
RA: Right atrium
RV: Right ventricle
RVEDd: Right ventricular end-diastolic diameter in diastole
RVEDV: Right ventricle end-diastolic volume
RVESV: Right ventricle end-systolic volume
RVFAC: Fractional area change of the right ventricle
RVGLS: Right ventricular global longitudinal strain
RVIT: Right ventricle inflow tract
RVSV: Right ventricle stroke volume
RVEF: Right ventricular ejection fraction
RWTd: Relative wall thickness in diastole
SV: Stroke volume
TAPSE: Tricuspid annulus peak systolic excursion
VO_2 max_: Maximal oxygen uptake
