# Supplementary material for: Moderately trained male football players, compared to sedentary male adults, exhibit anatomical but not functional cardiac remodelling, a cross-sectional study
Source: Cardiovasc Ultrasound. 2021 Nov 11;19:36. doi: 10.1186/s12947-021-00263-0 (PMC8582134; doi:10.1186/s12947-021-00263-0)

Additional File 1

VO2 max calculated from peak work load, compared with actual measurements in the athlete group, according to Brudin, pp 251-254 in Jorfelt, L and Pahlm O: Kliniska arbetsprov, Lund, Studentlitteratur 2013 ISBN 9789144083834).

Recalculate the different steps to a factor ”K” (for details, consult “Kliniska arbetsprov” page 253 eq. 3 and 8). K is additive and its components can be added. K1 is at steady state and K2 is the ensuing increase in load.

Calculations:

$K1=6*(100)^5$ according to eq 8.

$K2=(Wmax^6-100^6)/30/6$ according to eq. 3.

$K=K1+K2=6*(100)^5+(Wmax^6-100^6)/30/6$

Convert K to Wmax6´ according to eq 6.

$Wmax6' = (K/6)^{0.2}$

Enter K into eq 11 and calculate the comparable load at a 6 min steady state cycling, Wss.

$Wss = Wmax6'(1-0.2(1-50/Wmax6')^5)$ (males)

estimate max VO2 in eq 12 according to I Åstrand (Arbetsfysiologi. Norstedts förlag 1986).

$VO2 = 12.7Wss/BM +4.6$

The factor 4.6 is the basal metabolism per kg and ”BM” is body mass expressed in kilos.

This results in the following relationship between calculated and measured VO2.

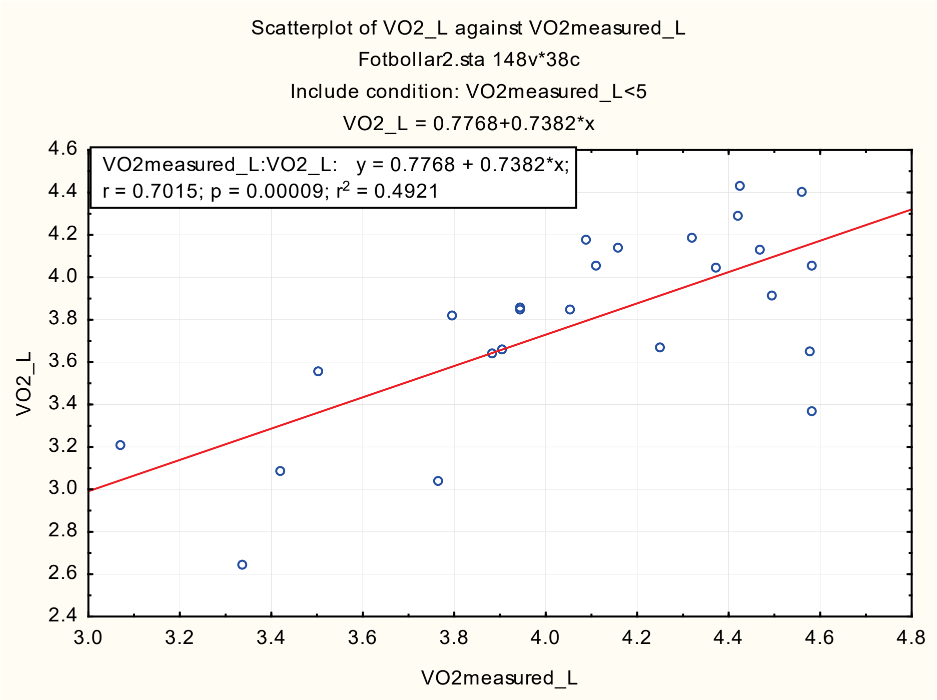

If adjusted for body mass, VO2 per kg relates better (0.96*VO2measured).

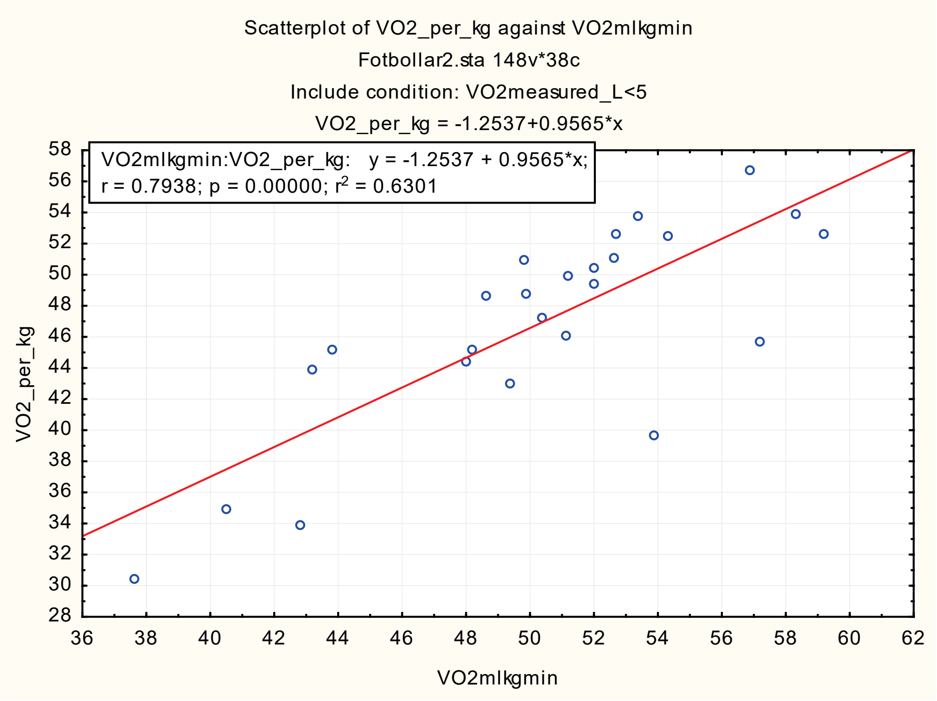

Supplement: Supplementary file 1 — Additional file 1. Computation of regression equation between measured VO2max and calculated VO2max, developed in the group of athletes. [file 12947_2021_263_MOESM1_ESM.pdf]
